# Differences in teletherapy and telecare?—Experiences of health professionals and patients with video communication in nursing, physiotherapy, occupational therapy, and speech therapy

**DOI:** 10.1177/20552076241301963

**Published:** 2024-12-05

**Authors:** Norbert Lichtenauer, Felix Schlachetzki, Inge Eberl, Annette Meussling-Sentpali

**Affiliations:** 1 64381 Deggendorf Institute of Technology (THD)/Faculty of Applied Health Sciences - Health Campus Bad Kötzting, Germany; 2 235827 Ostbayerische Technische Hochschule (OTH) Regensburg, Regensburg Center of Health Sciences and Technology, (RCHST), Germany; 3 235827 Ostbayerische Technische Hochschule (OTH) Regensburg, Faculty of Health and Social Sciences; Nursing Science, Germany; 4 Department of Neurology, TEMPiS Telestroke Center, Medbo Bezirksklinikum, 210419 University of Regensburg, Germany; 5 Catholic University of Eichstätt - Ingolstadt, 229957 Faculty of Social Work, Germany

**Keywords:** Teletherapy, telenursing, synchronous video communication, video consultations, guideline, recommendation for action

## Abstract

**Introduction:**

The spread and implementation of digital synchronous video communication in telecare and teletherapy has recently increased significantly, not just because of COVID-19 but also due to a global trend towards more digital healthcare services in the last two decades. This shift prompts the question of how digital telesetting differs from a face-to-face setting and which aspects are fundamental.

**Methods:**

As the first part of a mixed-method study, qualitative interviews (*n* = 20) were conducted from July 2021 to January 2022. Health professionals (*n* = 13) and patients (*n* = 7) from occupational therapy, physiotherapy, speech therapy, and nursing were interviewed. All interviewees came from Germany, Austria, and Switzerland. The results were categorized using structured content analysis.

**Results:**

Six main categories and 20 sub-categories were summarized, which can act as barriers or resources in a telesetting. Both sides described a high level of acceptance and approval of telesetting. Motivation and digital skills were of great importance. Furthermore, special features in communication and interaction were described, as well as changes in organizational procedures and a specific process flow in telesetting. Including relatives was more feasible, although several environmental factors should be considered.

**Discussion:**

A number of specific changes in a telesetting compared to a face-to-face setting show the need for a structured guide for interested parties. Appropriate basic principles must be taught in training and further education to support the spread of this new form of care. Furthermore, it is crucial to adapt the methodological and content-related aspects of telesetting and develop new approaches that specifically integrate audio-visual possibilities.

## Introduction

### Demographic changes are increasing the number of cases for healthcare services

Demographic change is considered a relevant fact in health policy and economics worldwide, especially in highly developed countries. It has far-reaching consequences for the care situation and the financing of services in the healthcare system.^1^ Society's aging due to too few births and people's increased life expectancy is triggering a dynamic for increased case numbers in the healthcare system. A further increase in the number of people needing care and therapy is predicted, with associated cost increases.^2,3^

#### Telemedicine for effective use of resources

Telemedical approaches in the healthcare sector, like telecare and teletherapy, have considerable potential to maintain or improve the safety, efficiency, and quality of care and to reduce healthcare costs.^4^ Modern internet-based communication technologies play a central role but are not yet routinely used.^5^ The COVID-19 pandemic also accelerated the development of digital interventions in outpatient healthcare settings^6^ and showed its benefits in healthcare.^7^ The format of video consultations is a promising way to meet patients’ needs, reduce healthcare costs, and ensure high-quality care in the long term^8,9^ or improve access to healthcare services,^10^ including for people with communication or cognitive impairments.^11^ In the best-case scenario, personal visits to medical staff or home visits can be saved, relatives can be more involved, and hospital admissions can be reduced.^12,13^ The potential savings from establishing digital care pathways in Germany are enormous. According to a 2022 study, the consistent implementation of 26 digital solutions could generate savings of up to €42 billion per year for the German healthcare system, with the most significant savings potential seen in online interactions at up to €12 billion per year.^14^

#### Telecare and teletherapy offer opportunities

Both telecare and teletherapy are seen as areas of application of telemedicine that share the ability to participate actively in the treatment of patients from a distance and are defined using information and communication technologies (ICT).^15,16^ A distinction can be made between synchronous, asynchronous, and hybrid services^17^ in telesetting. Synchronous services include a live exchange and direct communication as a central element. Asynchronous offerings are conveyed via various digital communication channels, enabling time-delayed editing of apps, audio, and video files. Hybrid offerings include a combination of synchronous and asynchronous offerings, often in combination with a face-to-face setting.^17,18^ Synchronous video communication is used most frequently^18,19^ and should be seen as an extension of the possibilities to get in touch with patients.^20^ Applications for teleintervention in care primarily include counselling and instructing care recipients and their relatives, prevention and health promotion, monitoring vital data, and information exchange between professionals.^21^ Additional motor, communicative, and cognitive treatments are carried out within teletherapy.^22^ Other aspects include diagnostic activities and aftercare.^19^

#### Aim of the present work

The research objective of this study is to develop a recommendation for implementing video communication in telecare and teletherapy, as there are currently no guidelines and standards for teleinterventions.^19,23,24^ The aim is to record the experiences of healthcare professionals and patients from the fields of nursing and therapy to consider possible special features in communication, interaction, and the framework conditions in the future. For many healthcare professions, video consultation is still an entirely new way of working carried out without guidelines, which has often led to barriers in the past.^25,26^

## Methods

### Literature search

A sensitive literature search was conducted using the German Network for Evidence-Based Medicine recommendations (www.refhunter.org).^27^ The search was operationalized using the PICO scheme and aimed at existing guidelines and recommendations for patients and health professionals on synchronous video communication in care and therapy. It was conducted in the medical and health science databases MEDLINE (via PubMed), CINAHL Full Text (via EBSCOhost), Cochrane Library, OTseeker, PEDro, speechBITE, PubPsych, and the cross-sectional database Web of Science.

### Research questions and objectives

The research question was “How do health professionals and patients from care and therapy experience synchronous video communication?”. The following secondary questions were also of importance:

- What communicative and interactive characteristics are perceived in synchronous video communication?
- Which conversation techniques and strategies are used according to the health professionals?
- How should the environmental factors be designed for synchronous video communication?
- What are the advantages and disadvantages of synchronous video communication?
- What other aspects are of great importance during synchronous video communication?

The aim is to develop a guideline for further establishing synchronous video communication in teletherapy and telecare.

### Research design

**Figure 1.**
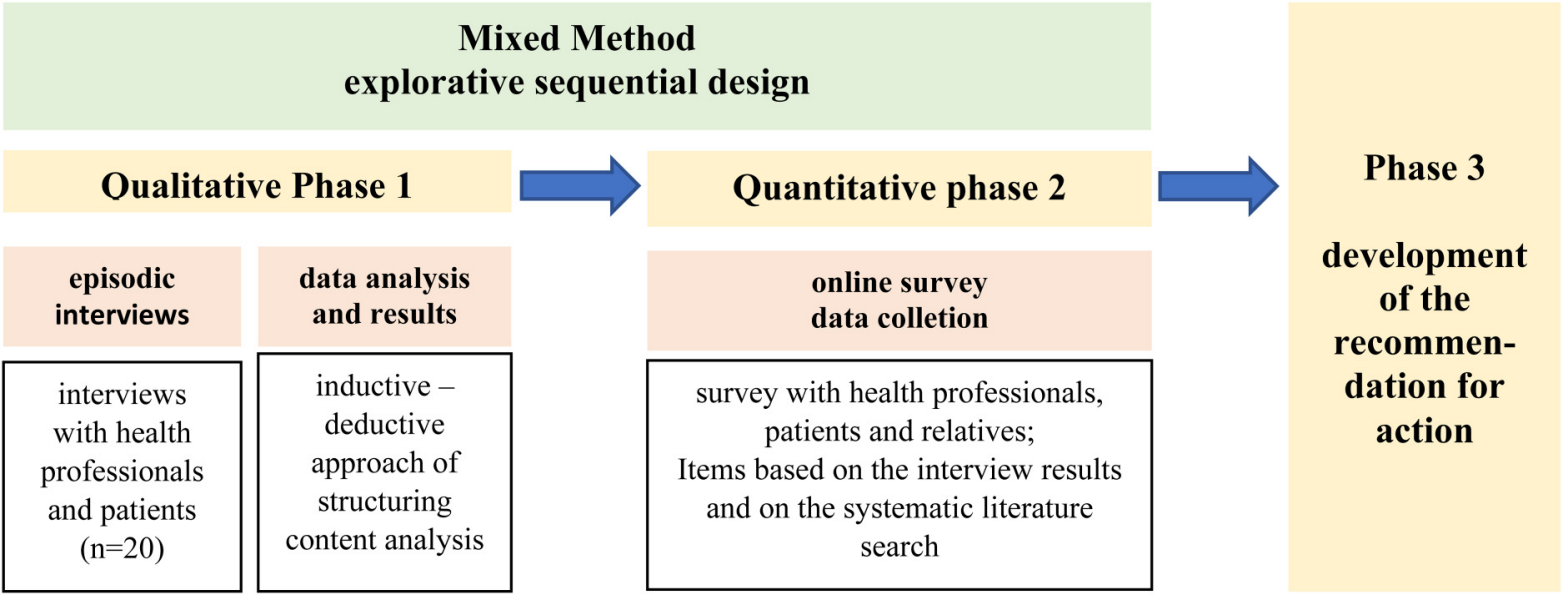
Illustration of the mixed methods approach; own illustration.

A mixed methods approach was chosen because it combines qualitative and quantitative research approaches,^28,29^ which seemed appropriate for the research subject (Figure 1). The preliminary results from the hypothesis-generating interview survey with a small sample have been completed. These will subsequently be validated and expanded using a questionnaire with more cases to develop the recommendation for action. The research questions are addressed in the triangulation of a systematic literature analysis, a qualitative interview survey, and a final quantitative questionnaire survey. This approach enables a comprehensive examination of the study subject using different perspectives and research approaches.^30^ Within the mixed methodology, the explorative sequential design was chosen. In this design, the two sub-studies are carried out one after the other, and the results of the first sub-study also influence the subsequent survey.^31^

### Ethics and data protection

To comprehensively consider ethical and data protection challenges in the research project,^32^ an ethics vote was submitted to the Ethics Committee of the University of Regensburg in advance of the survey and approved (No. 21-2451-101). The study was registered in the German Register of Clinical Trials following the positive ethics vote to enable transparency and avoid duplicate data collection (DRKS ID DRKS00025771).

### Method of data collection

The episodic interview was chosen for the Phase 1 qualitative sub-study (Figure 1) because it generates open narrative accounts of experiences and enables definitions and correlations of terms.^33^ The interviews were conducted using a structured guideline developed based on literature research and a system for creating questionnaires.^34^ No copyrights were involved in creating and using the questions in the interview guide. The interview questions were not shared before the interview, and the guideline was not validated. A cognitive pre-test of the questions was carried out to check linguistic and content-related items for comprehension.^35^ Test interviews were also conducted with two physiotherapy and occupational therapy therapists.

### Sample and implementation

After defining the inclusion criteria (Table 1), the first health professionals and patients were selected using theoretical sampling. After a preliminary telephone interview or written information by e-mail, they were interviewed about their experiences with synchronous video communication in teletherapy and telecare. Snowball sampling was used later in the survey.^36^ A call for participation was also made via the relevant professional associations, specialist journals, social media channels, and personal networks.

**Table 1.** Inclusion criteria sample (*n* = 20).

Criteria for health professionals and patients
Age of majority
Ability to give consent
sufficient language to be able to conduct interviews in German, both reciprocally and expressively
Experience in conducting synchronous video communication in the areas of care or therapy9
Additional criteria for health professionals only
Training in the fields of occupational therapy, speech therapy, physiotherapy, or nursing
In addition to direct work with patients, activities also include the organizational level in the facility, e.g., managers or specialist managers

Twenty interviews (health professionals: *n* = 13, patients: *n* = 7) were conducted exclusively by the first author of this study after comprehensive clarification using informed consent.^37^ The interviewer had prior knowledge of the topic due to his experience conducting telesettings as an occupational therapist. Recommendations for conducting guided expert interviews were followed.^38^ The data collection lasted from July 2021 to January 2022 and covered the period affected by the pandemic restrictions in Germany. All interviews were conducted online without prior acquaintance, and no other person was present.^39^ Before the interview, the author gave a brief personal introduction and repeated the objectives and background of the study. During the interviews, notes were taken. The audio track was recorded via the video conferencing tool and then transcribed based on the rules of Przyborski and Wohlrab-Sahr.^36^ The interviewees did not proofread the transcripts. According to the principle of theoretical saturation,^36^ interviews were collected until redundancy in the data became apparent. No interviews were canceled during the survey or refused to give answers. No repeat interviews were conducted.

### Qualitative data analysis

The analysis was done with MAXQDA 2020®, using the inductive-deductive approach of structuring content analysis from Kuckartz.^40^ After initial text work, preliminary deductive main and subcategories were created using the interview guide. The categories were supplemented and specified by inductive codes through an initial coding of six interviews. Subsequently, the author coded all interviews with the revised categories.^40^ To enable a collaborative evaluation and create intersubjective comprehensibility, selected passages of the data material were discussed in interpretation groups with other healthcare researchers.^41^ In addition, an intercoder reliability check was carried out in MAXQDA.^42^ For this purpose, two interviews were intercoded by two experts conducting qualitative research on similar topics. The interraters received interviews in which the researcher assigned segments to a main category in advance. The interraters then had to assign the pre-defined segment to corresponding sub-categories and could also leave comments regarding the main category. According to Kuckartz and Rädiker, this procedure is recommended to calculate a randomly corrected kappa according to Brennan and Prediger.^43–45^ Since both interraters reviewed different interviews, a common kappa value was not possible, and a mean value was formed from the two kappas, corresponding to the correct procedure according to Kuckartz and Rädiker.^45^ The Kappa K_n_ was 0.75, which, according to Landis and Koch^46^ corresponds to substantial agreement and according to Altmann^47^ to a good agreement. This was followed by an exchange with the interrater persons, and a revision of the category system followed.

### Quality criteria

Since a mixed methods survey combines qualitative and quantitative research methods, the quality criteria of the respective approaches must also be considered.^48^ The four criteria of trustworthiness, transferability, reliability, and confirmability are central to qualitative research.^48^ Furthermore, in the process, the appropriateness of the subject matter, empirical saturation, theoretical penetration, textual performance, and originality were comprehensively considered as descriptions that characterize quality.^49^ In addition to the quality criteria mentioned, the international recommendations for qualitative (mixed-methods) research of the COREQ,^50^ SRQR,^51^ and MMARS^28^ Guidelines and the CROSS^47^ Guideline will be adhered to for the quantitative design.

## Results

### Socio-demographic data

Thirteen interviews with health professionals and seven patient interviews were conducted with people from Germany, Austria, and Switzerland (Tables 2 and 3). A total of 826 audio minutes of interview material was collected (MV 41.3 min/interview).

**Table 2.** Participating health professionals (*n* = 13) and patients in the interviews (*n* = 7)/MV = mean value/R = range.

Group	Profession (1)/background (2)	Age	Total work experience/experience with video communication (1) units with video communication (2)
(1) Total health professionals (*n* = 13)	Physiotherapy (*n* = 3) Occupational therapy (*n* = 4) Speech therapy (*n* = 4) Nursing (*n* = 2)	44 years (MV) 31–64 (R)	20.4 years (MV)/1.37 years (MV)
(2) Total patients (*n* = 7)	Physiotherapy (*n* = 4) Occupational therapy (*n* = 2) Speech therapy (*n* = 1)	39 years (MV) 23–62 (R)	14,33 units (MV)

**Table 3.** Categories from the structured content analysis of the interviews (*n* = 20).

Categories	Subcategories
Person	First experiences
	Digital skills
	Motivation
	Patients
	Health professionals
Communication	Nonverbal communication
	Paraverbal communication
	Verbal communication
Interaction	Relationship
	Physicality
	Relatives
Process	Structuring
	Contents
	Quality aspects
Organization	Internal processes
	Alternative offer
	Economic efficiency
Environment	Spatial aspects
	Technical aspects
	General conditions

### Categories and subcategories

The interview statements were subsumed into six main categories and twenty subcategories (Table 3).

### Person

Overall, *the initial impressions* of video communication showed few differences from the face-to-face setting. “I can't think of any major differences per se compared to face-to-face physiotherapy, I would say it is very targeted, but it also depends entirely on who you are doing the physiotherapy with and it can be just as targeted in a face-to-face setting” (Patient IP^1^18, 42). Overall, telesetting was highly accepted and was described as a practical and flexible way of exchanging information. “I think it's brilliant technology like this; I mean, you could be on the moon, and communication could take place” (Patient IP16, 103). This also resulted in new learning opportunities. “So, for me, it was a gain and a learning experience; I don't think I've learned so much for ages” (Health Professional IP7, 74). In the first units of the telesetting, there were also reports of personnel, technical and organizational hurdles. “The first hour was a bit restless, a bit of jumping back and forth with the technical things, a bit of a mess” (Health Professional IP9, 16).

*The participants’ digital skills* were seen as both a resource and a barrier and were highly important for telesetting. With previous experience and technical expertise, telesetting could be used well and quickly, and younger people, in particular, had fewer problems. “I think it makes a difference whether the person receiving video therapy is technically experienced” (Health Professional IP10, 16). “So it was very different, so we had […] different age structures; the younger ones often found it a bit easier to use this technology” (Health Professional IP6, 18). A lack of digital skills was the main barrier to implementing telesetting. “The technical compliance, whether people can really get it right, so to speak, are the barriers” (Health Professional IP1, 26).

The great importance of *motivation* was also discussed. It could be carried out successfully if this was present for a telesetting. “If the patients were motivated, we always found a way to offer it” (Health Professional IP10, 18). Personal attitudes and the recognition of individual advantages were important. “I am a whole lot more open, I can detach myself more from such things and like to try them out” (Patient IP3, 72) and „also quite comfortable, I say living room atmosphere because you can do it quite easily from home“ (Patient IP18, 10). Extrinsic motivations were also described, such as managers wanting to inspire employees. “So for me personally it was an innovation, I wanted to offer it, for me in my position at work it was also a pioneer, a role model” (Health Professional IP10, 24). However, a lack of motivation to engage with the technology and skepticism about this form of interaction were obstacles. “The employees were all very skeptical, we're not going to do that, how is that supposed to work” (Health Professional IP4, 20).

In addition, *specific personal aspects of patients* for telesetting were described that had to be taken into account. The ability to perceive the body was seen as an essential factor to avoid drawing false conclusions for counseling and treatment.

If someone has poor body awareness […] then video communication makes little sense in that case because people are more or less left out in the cold with the exercises, they don't even know what to do, they probably do it wrong, they document it wrongly so that the wrong conclusions can be drawn and you may end up training completely past the problem. (Patient IP19, 68)

The individual state of illness or health was also considered decisive for the feasibility of telesetting, and the need for physical contact represented a natural limit. “The disorder must fit, so of course it doesn't work for everyone” (Health Professional IP9, 22) and “apart from the situation that we can't go hands-on and have to think very clearly about which clients we can offer video therapy to” (Health Professional IP10, 52). Sensory, physical, and cognitive requirements for telesetting were also mentioned. “Maybe you can say that it is contraindicated […] if he can't realize what you actually want, which means that he doesn't understand it, that he is insecure” (Health Professional IP1, 48). In addition, self-management skills were essential for the process, and personal responsibility for content was emphasized. “Then the patient must also be able to carry out the exercises themselves actively” (Health Professional IP5, 52), and “The advantages were definitely that the patient really became more aware that he really has to work on it” (Health Professional IP5, 48).

There were also *specific personal aspects for health professionals* mentioned for a telesetting. Individual attitudes to how learning experiences are handled were important. “First of all, the willingness to try it out at all to gain the experience” (Health Professional IP14, 43). There should also be a reflection on whether the telesetting fits in with the previous professional understanding of the role and the way of working. “How you see yourself as a therapist, so I already know colleagues who really like coaching, it's not so much me now” (Health Professional IP2, 70). Professionals should increasingly adapt their behavior in the telesetting and radiate calm and security in contact. “The most important thing for me was to convey a sense of security and calmness with those affected, i.e., this target group” (Health Professional IP20, 10). Overall, observations by professionals in telesetting were more intensive than in a face-to-face setting, which required more concentration and effort.

At least I had the realization that you have to be even more present and even more in the observation and in the perception, because you only see a part of it and don't have the whole body language […] so I personally found it more exhausting, because you always have to be 100% present. (Health Professional IP20, 14–18)

### Communication

Communication was essentially identical to the face-to-face setting, although specific changes in telesetting were mentioned. In *nonverbal communication*, the increased use of gestures and facial expressions was described, some of which were also verbalized. “I have started to gesticulate more, thumbs up here and also to give more feedback using gestures and facial expressions” (Health Professional IP11, 35) and “You have to verbalize your gestures and facial expressions, everything you see is important” (Health Professional IP7, 56).

Furthermore, the significance of *paraverbal aspects* was discussed more than in a face-to-face setting. The pace of speech should be slower, and voice modulation should be more targeted. “That you have to pay attention to the tempo, so to speak, and also to the pronunciation, so rather a slower tempo” (Health Professional IP20, 40). They also spoke a little louder, which could also lead to vocal complaints. “I remember that it affected my voice because you still speak very loudly, so I'm talking louder again now […] you're shouting a bit” (Health Professional IP9, 14).

In addition, *verbal communication* showed some specific characteristics compared to the face-to-face setting. The general importance of communication grew, and it was considered the primary method in telesetting. “Video lives from communication; the main thing we have here is communication” (Health Professional IP14, 51). Compliance with conversation rules was described as more difficult due to delayed audio transmissions, more interruptions, and fewer speaker changes compared to a face-to-face setting.

So the communication is a bit different for me in that […] it is difficult to adhere to the rules of conversation, so you simply have less on the screen when the next person finishes their sentence, and I can say something about it” (Health Professional IP11, 13) and what is, of course, a difference, and what may be due to the slight delays I think, is this turn-taking that means that you still switch back and forth more quickly in a real conversation. (Health Professional IP9, 64)

Overall, the language was described as less formal and more personal, “the type of language was actually different too, so it was less formal” (Health Professional IP10, 40). Small talk hardly changed and continued to be used intensively, especially to capture everyday life. “Personally, I also think it's important that you don't discard familiar phrases that you usually use in everyday life […] I always find a round of small talk at the beginning important in the sense of check-in, check-out” (Health Professional IP6, 60), and “that's where you get interesting insights that help you […] for example, where you carefully give structural hints about the daily routine” (Health Professional IP14, 85). The questioning behavior was described as very similar to the face-to-face setting, with more personal questions being asked. “In my experience, it's actually no different to face-to-face therapy, so they bring their concerns, bring their questions, no difference” (Health Professional IP8, 74), and “To ask us very personal questions about your children's development, which they often refrain from doing when the children are standing next to them” (Health Professional IP10, 36). It was also important for self-management, personal responsibility, and self-efficacy. “I always do that again to say whether he has understood everything correctly, whether he still has questions […] and that he can then continue this in some form in self-management” (Health Professional IP5, 26), and “I think you gain a lot of self-efficacy through it and you realize that you have to tackle the problems yourself in any case” (Patient IP18, 46). The use of conversation techniques was described as quite similar to presence, “so I actually greeted my patients as I always do […] the process and the way I communicated felt just as natural in the end, no special technique” (Health Professional IP8, 72), although active listening, action-accompanying speech, and feedback discussions were sometimes considered more important. “So active listening plays a very special role again” (Health Professional IP6, 28), and “so that it's not irritating, I verbalized everything, so I also said I'm going to go down quickly and pick this and that up from the floor […] so that it's not seen as disrespectful or […] as a lack of interest (Health Professional IP7, 56), and „the patient also understood this when working and looking in the feedback discussion“ (Health Professional IP5, 28). The communication channels were extended by the chat functions of the video software, emails, and messenger services, and the exchange was more regular.

When we offer video therapy, it either happens afterward that you then write an email […] or patients can then also ask their own questions beforehand in the chat […] they can also use additional tools that they might not have used before. (Health Professional IP10, 32)

### Interaction

The interactions were sometimes described as changed and sometimes identical to face-to-face therapy. Establishing a *trusting relationship* was similarly successful in the telesetting as in face-to-face therapy. “So I don't find it difficult to build up the same bond with the patient-therapist as in face-to-face therapy, I think it works very well” (Health Professional IP11, 17). Eye contact, on the other hand, was made more difficult for technical reasons, which was often discussed to avoid irritation during interactions. “You either look very directly into each other's eyes, and that can also be unpleasant, or when someone looks away and misunderstands […] such interactions have to be communicated” (Health Professional IP8, 68). Showing empathy was considered more difficult compared to the face-to-face setting, and emotions could not be felt either. “This empathy in particular, you can also bring that into video therapy, feeling into the patient through the camera, that's possible, but it's not comparable to direct contact” (Health Professional IP2, 52) and “I also find it a hurdle to feel less or nothing at all” (Health Professional IP8, 44). The familiar setting of their own home often seemed to create a sense of security and made patients more open and talkative. “It was a more informal approach, because that's my guess, the environment they were in was familiar to them and natural and cozy at home […] takes away a bit of shyness, makes them more talkative” (Health Professional IP14, 83). However, the danger of leaving one's living area less due to the telesetting was also discussed here. “But it can also lead to people leaving their own home less - that would be a disadvantage” (Patient IP3, 46). More attention had to be paid to intra-family relationships, especially in the case of dependency relationships or expectations of success from relatives. “You really have to check whether things are going well emotionally and socially between them […] although you mean well as a therapist, a lot can go wrong socially” (Health Professional IP14, 39).

In the *area of physicality*, some negative aspects of the interaction became clear. For example, a loss of contact was described due to the lack of physical presence, which was described as a clear difference in presence. “The biggest difference is the lack of personal contact, which […] should not be underestimated” (Health Professional IP1, 36). The limited observability of body signals was often described as a further disadvantage of the interaction. “The body signals, whether the patient is agitated, tense, their posture, their smile […] just these classic ones […] that is always easier to assess in direct contact” (Health Professional IP2, 38) and „I can't judge what is happening below the upper body […] that is perhaps a disadvantage compared to face-to-face therapy“ (Health Professional IP10, 46). The aura and charisma of the other person were hardly present and could create distance. “We all have our aura around us, we all have our charisma; however, you want to express it, and a lot of that is lost with video; it's just not there” (Health Professional IP4, 54). Interacting with each other was also described as challenging and sometimes perceived as less intense. “It's also another hurdle because when someone comes to my practice, we're two people in one room, so I'm a face on a screen […], and I think people interact a bit better with people than with screens” (Health Professional IP15, 30). Overall, interactions were often more focused on content in the telesetting. “I would say that especially with Name2 […], much more attention was paid to my problems” (Patient IP18, 22). It was important to initiate telesetting interactions and activate the other person.

Yes, I always find it very important to activate the other person at this point, i.e., to explicitly ask them to do so again and again and […] as a speaker, you are even more challenged to somehow (…) enter into interaction with the other person and to stimulate this interaction from the other side. (Health Professional IP6, 26)

In some cases, the interaction was also perceived as lonely and sterile, which could lead to the rejection of telesetting over time. “It did get lonely and sterile at some point, so it was good at first, and at some point, nobody wanted it in the long term” (Health Professional IP8, 80). The inability to intervene on-site could also lead to a loss of control and anxiety among health professionals. “In a way, I have a loss of control over the patient because I can't influence what happens on-site” (Health Professional IP10, 46), and “What I always experience is the fear that you don't know what to do […] that you can't restrain the child, can't motivate them, and of course, it's more difficult via video” (Health Professional IP7, 36).

The *participation of relatives* in telesetting was perceived as more manageable and more substantial compared to the face-to-face setting and was considered a great advantage. “I noticed that in the last video therapies, parents are basically sitting in and the parents act as co-therapists, a very important and really great point” (Health Professional IP14, 37). In addition, more information was exchanged, and relatives were made more aware, which could also shift professionals’ focus towards relatives. “The communication was actually more, so the content flowed more, but the child itself or the patient itself was often left out” (Health Professional IP9, 56), and “What also came from the feedback was that they now understand the clinical picture of dementia better thanks to this regular support” (Health Professional IP20, 56). It was difficult when relatives were pushed to the fore. “Sometimes we had to slow down the relatives we brought on board a little because they put themselves too much in the foreground” (Health Professional IP2, 40). The understanding of the role of relatives among professionals also changed. In telesetting, they were sometimes described as assistants who compensated for implementation and communication problems, helped with the preparation, and had a calming effect on patients. “In the interaction with the relatives, too, to carry out this therapy together with the woman […] there is a lack of verbal communication in some parts (with the patient) and from the context we always carried out the exercises in threes […] she was partly my hand, my assistant” (Health Professional IP5, 30), and “the parents participate in the sense of printing out documents that should then be looked at like these little cards that should then also be colored in and cut out” (Patient IP17, 39). A telesetting was also described as relieving the burden on relatives due to the distance saved. “In terms of time, of course, it's a huge advantage because you don't have to drive to the practice to see the patient […] you don't need a relative to drive either” (Health Professional IP2, 52).

### Process

Some changes to the face-to-face setting were described *in the structuring of the process*. Preparation, follow-up, and data protection required more time and resources in the telesetting. “That meant a lot of preparation for us at the beginning, that meant obtaining consent, a lot of e-mail correspondence, a lot of preparation in the form of design, new data protection, compliant platform” (Health Professional IP10, 12). The necessary technical instruction was seen as the responsibility of experts and managers. “Of course, you always have a responsibility not only for your own content but also to a certain extent if you are the organizer for the technology” (Health Professional IP6, 26). Appropriate training was essential here and conveyed a sense of security, although it was challenging to communicate at a distance. “We did staff training with regard to the technical care of the patients” (Health Professional IP10, 12), and “Yes, it was really difficult at times […] especially when there were problems afterwards and solving them at a distance” (Health Professional IP6, 20). Diagnostics were described as more difficult, and standardized instruments were recommended wherever possible.

You also don't know how to design an examination like this, or an anamnesis via video, if I don't really have the person right in front of me […] I have a list of questions ready […] where I say ok, I can ask certain things, they should always be the same in a standardized way. (Health Professional IP15, 16–20)

On the other hand, a medical history and everyday orientation were easy to integrate into a telesetting. “I can do it well when I take a medical history to get started, to ask about certain things” (Health Professional IP4, 26), and to “Simply (gain) insight into the home environment” (Health Professional IP7, 80). The rough sequence of the tele-units was quite identical to the presence.

A brief setting of goals for today's lesson […] then it really goes into the exercise sequence […] both with adults and with children […] so nothing really changes in the process whether it's face-to-face therapy or video therapy, it's always the same process. (Health Professional IP10, 28)

In the telesetting, however, specific information was given about the process and technical aspects.

That you have a certain amount of certainty about how long it will actually take, pointing out to the patients that you can, of course, also see into their private environment through the camera's view, simply informing them. (Health Professional IP2, 22)

Tasks were more pre-structured, and improvised action was more difficult. “There is simply a clear structure behind the whole thing (exercises), a folder for the video therapy so that he always has everything to hand and this material is always to hand in our practice” (Health Professional IP2, 36). The achievement of goals was communicated very clearly.

Online, you always have to say clearly beforehand, this is what we want to achieve today, and yes, and then we'll see what happens, but the goal setting at the beginning is very important, so that means the patient must always be clear what are we doing now. (Health Professional IP1, 30)

*Differences in the content* compared to a face-to-face setting were also discussed. These resulted from a lack of materials or methods unsuitable for telesetting, so that content was often considered and adapted together. “Above all, I don't have as many opportunities to treat the patient at home, I have other material” (Health Professional IP5, 48), and “Yes, and then we thought about what we could do via video […] we just came up with yoga, she does something before I do it after […] yes, we did that too” (Patient IP13, 11). Prevention and counseling services were also expanded. “That you also go into counseling and prevention, in fact, that was not such a big request in our practices before, and that has actually grown” (Health Professional IP10, 32). The materials and methods used were often taken from everyday situations and were intended to provide haptic stimuli. “Because we were simply able to dissolve the washing situations, my son didn't even notice his therapist at that moment, he was sitting on the floor stretching out and growling to himself […], and the laptop didn't matter anymore” (Patient IP3, 52), and “I find that if you describe it superficially, the haptic, that several senses are nevertheless addressed […] that the patient actually has something in his hand and can feel that it has to do with the therapy” (Health Professional IP2, 30). In some cases, materials were sent or obtained independently by patients, and the use of videos was described. “So we also brought exercise equipment home to the patient […] the patient needs dumbbells […] then we brought it to the patient's front door” (Health Professional IP2, 28), and “this also taught us how we can motivate the patients with additional videos, for example, by additionally writing down instructions for action” (Health Professional IP10, 32).

*In terms of quality*, a successful and high-quality telesetting was primarily associated with functioning technology.

Yes, the technical story was the most important thing, that at least that's the basic prerequisite, it has to work, otherwise, if it's always running around, then it's no fun for the children, the parents and of course not for us either. (Health Professional IP9, 18)

Furthermore, an increased focus on everyday life and a successful goal orientation in telesetting were mentioned as positive quality features.“A big advantage was that it took place in our home because we wouldn't have had a home visit, yes, so we could use the bathroom to deal with the washing situation directly and not somehow with a washing bowl in practice, which doesn't really make any sense because it's not the place where the conflicts take place” (Patient IP3, 46), and “When patients make progress that's always the case […] it's very goal-oriented and should improve the symptoms” (Health Professional IP11, 69). Patients were often more active in the telesetting, and self-efficacy was supported. “I would say that, as a rule, this online setting helps to improve self-efficacy because […] it is clear from the outset that it is not possible to do the exercises together” (Patient IP18, 48). Furthermore, the telesetting made complying with safety and hygiene measures easier. “I'm in quarantine and would have to cancel twice now for two weeks, or I've just got a cough. Can I come into the practice then? We actually say no for safety reasons” (Health Professional IP9, 70). Negative quality experiences were limited equipment and technical problems. “The equipment of the households, i.e., the spatial environment of the clients, those were my limits, so if the child has no glue, then it has no glue […] or because the printer was broken and you send something” (Health Professional IP7, 48). Some patients had problems with their working posture, meaning telesetting was sometimes rejected. “To say to my daughter she found it difficult at first to find any communication because she didn't know how to act” (Patient IP17, 13). Quality development is expected through research and new developments. “We should definitely continue to investigate whether video therapy makes a difference compared to face-to-face therapy” (Health Professional IP10, 52), and “I believe that it will also improve in terms of quality because you simply have to think about the material that suits the individual disorders” (Health Professional IP11, 77).

### Organization

Changes in the *internal organization* were reported as a result of telesetting. In the area of work organization, work intensification and reduced relief times were described, and usual working hours were shifted. “It is both a blessing and a curse […] in practice, it often leads to a concentration of work […] you can just put appointment after appointment after appointment and the relief times in between are eliminated” (Health Professional IP6, 50), and “So I was quite flexible in terms of time […] I even did therapy on Sundays” (Health Professional IP7, 44). Making appointments easier was described. “Cancellations at short notice were quite common due to the quarantine situation, but it was also possible to quickly offer another appointment, so there was a lot of flexibility on both sides” (Health Professional IP10, 40). The flexibility of person and place was also seen as a great advantage, especially when looking for specialists.

For me, this is an opening of boundaries that we didn't have before, that I can no longer just work offline from person to person, but that it is now independent of place, time, and space, and I can also get in touch with people who are now not in my circle. (Health Professional IP7, 10)

Time advantages were mentioned in terms of reduced travel distances and better compatibility of appointments with everyday life. “You also save time […] you can save the trip to the practice” (Patient IP12, 40), and “That is, of course, a clear time saving, so we were able to schedule the appointments so that they took place directly after school” (Health Professional IP6, 38). Furthermore, a simplification in inter- and intradisciplinary exchange through telesetting was reported. “It simply expands the possibilities for communication, even at short notice, so if you want to exchange opinions on a topic at short notice, it's really great to just send someone a link and say, come let's meet” (Health Professional IP6, 64).

Telesetting was also seen as a low-threshold *alternative* care option in illness and crises, which enables further support. “The advantages, especially during the first lockdown, when we didn't yet know what the pandemic would bring […] it remained a good option […] that the therapy could still continue in this form” (Health Professional IP5, 48), and “For patients who were afraid, panicked or so depressed that they couldn't come to the practice, they still had this therapy session” (Health Professional IP7, 80). Telesetting has helped to counteract social isolation, prevent hospital stays, and provide continuous support for chronic illnesses. “Because you can also clearly say that you now realize that this can also prevent social isolation or contribute to maintaining contact with relatives” (Health Professional IP6, 70), and “So I think for highly acute patients it can be a way to be in contact […] it can hold something […] I think this can prevent many a hospital stay” (Health Professional IP8, 46), and “I think it makes a lot of sense for chronically ill children […] if she has bad phases, then we really would have had to cancel week after week […] which is a real shame because then she doesn't get to enjoy the appointments and it's difficult for the practice with all this canceling” (Patient IP17, 79). Telesetting was described as a substitute for home visits, particularly in underserved regions. “So it is a substitute for the home visit” (Patient IP3, 46). The combination of remote and face-to-face settings was recommended, especially in phases of aftercare or consolidation. “So that you don't perhaps meet physically so often and use it in between, for example, if you provide closer support […] as a combination, I would simply see it as good” (Health Professional IP20, 74), and “To provide support in the phases where it is about consolidation, so that I have to carry out fewer therapies on-site […] pattern changes take half a year, so I don't have to call the patient into the practice every two weeks” (Health Professional IP14, 101).

*Aspects of efficiency* that changed in telesetting were also described. The expansion of the clientele was seen as advantageous, as telesetting provided economic security, minimized risk, and compensated for absences. “The field of work it even broadens this field of work in principle I can serve a different kind of clientele” (Health Professional IP15, 14), and “I wouldn't want to miss it anymore, so especially as far as absences, spontaneous absences are concerned, absolute profit, risk minimization for the entrepreneur, absolute profit” (Health Professional IP14, 99). The proportion of telesettings varied greatly during the survey period but declined after the end of the pandemic. “The number of patients has decreased significantly I only have two patients myself now I used to work from home two days a week because I had online therapies all day” (Health Professional IP10, 50). In the future, telesetting will be seen as a new care pathway, and more people will have the necessary technology and skills. “I am absolutely convinced that this will be normal at some point, that there will, of course, be on-site treatments, but that it will simply emerge as another pillar” (Health Professional IP1, 54), and “I think that the technology will progress quickly and that more and more people will have access to this technology” (Patient IP16, 101). Concerns about the lack of financial support for investments and falling treatment prices were expressed. “I think it would be important to emphasize that we have regular funding, also for things like this […] we need funding opportunities to establish something like this” (Health Professional IP6, 76), and “If videotherapy is really to become more widespread, the lobbies must take good care that we are not pushed down even more in our prices” (Health Professional IP8, 90).

### Environment

In the area of environmental factors, changes in *spatial aspects* were described. The home environment was discussed in detail in advance and should offer sufficient space. “They should have room to take five steps, I have to clarify that” (Health Professional IP4, 68). Creating a quiet environment in the telesetting was essential for building trust, data protection, concentration, and a working atmosphere. “Yes, the protected atmosphere is very important […] in addition, an undisturbed room, this quietness” (Health Professional IP20, 44), and “I think […] to look for a rather quiet environment, that you are simply not disturbed by background noise, that you can also concentrate on the conversation” (Patient IP12, 52). Furthermore, increased distraction factors were reported for patients in the home setting. “I think the distraction factors are higher in the home” (Health Professional IP6, 36). A professional setting was discussed for professionals, which can be created by tidy rooms, professional clothing, and a reflective working attitude. “So from a therapist's point of view, it really makes sense to do it in the familiar practice rooms, which has such professionalism, I think work clothes are cool […] so to give it a value” (Health Professional IP2, 30).

The high importance of *technical aspects* was also mentioned. High usability of the software was crucial for a functioning telesetting and the reduction of barriers. “if you decide on this method of video communication, you also decide on a tool that is as inclusive as possible and allows many participants on board” (Health Professional IP6, 26). Modern hardware and a stable internet connection were prerequisites and discussed in advance; external devices were recommended. “A stable internet connection […] maybe you can summarize it, a reasonable technology, that we then also switched to a headset and microphone […] yes, the technical story was the most important thing” (Health Professional IP9, 18). Telesetting was rejected if the technology was too unstable. “Of course, it is also important for me that the technology works, it is very unpleasant […] unsatisfactory if something doesn't work” (Patient IP16, 25). The advantage was that digital devices and skills could be used in other areas of life. “We used the video again to communicate with friends or acquaintances, so we didn't call, but used the video” (Health Professional IP8, 22). Increased media consumption and an abrupt end to the telesetting session were cited as technical disadvantages. “Another disadvantage is that you then tie the children even more to the screens” (Health Professional IP2, 54), and “this abrupt departure, leaving the session […] also leaves me with very uneasy feelings” (Health Professional IP8, 50).

Concerning the importance of framework conditions, the specifics of telesetting should be considered in future training, especially in systemic and multi-professional work. “And of course it must also […] be included in the curricula […] it must also be addressed, when can I do this, how do I involve patients […] how do I involve relatives, how can I involve therapists, in other words always implement multi-professional work in the team” (Health Professional IP6, 80). Furthermore, an uncertain legal environment regarding billing, documentation, indications, and diagnostics was discussed, which should regulate processes sustainably and substantially.

So what is possible in terms of framework conditions are we allowed to do diagnostics first yes or no online […] that must actually come from the health insurance companies I think they must also think about possibilities of digital signatures, possibilities of documentation. (Health Professional, IP10, 52)

## Discussion

The overarching research question addressed the subjective experience of healthcare professionals and patients when implementing synchronous video communication in care and therapy. The results show some specific changes in telesetting compared to a face-to-face setting.

On the one hand, communication and interaction were described as very different in some areas compared to face-to-face settings. Furthermore, important changes in the therapeutic process, the organization, and the framework conditions of a telesetting became clear.

In the first question, the subjective initial experiences of video communication in telesetting were interesting. In their initial experiences, the professionals and patients surveyed unanimously emphasized high satisfaction and acceptance of video communication in telesetting. Above all, individual advantages were mentioned, particularly the possibility of low-threshold use. Furthermore, telesetting was seen as a simple and convenient way to communicate with one another. These statements on satisfaction and acceptance were in line with earlier studies on tele-interventions in other professional fields.^52–56^

Another essential question concerned possible changes in communication and interaction in telesetting. Many similarities to the face-to-face setting were described in both communication and interaction, and it was shown that much of what is considered necessary in a face-to-face therapeutic exchange also applies to telesetting. However, communication's importance grew compared to a face-to-face setting, and specific changes became apparent, for example, in the use of many different communication channels due to technical possibilities. Furthermore, non-verbal communication was sometimes additionally verbalized, and paraverbal communication aspects such as the pace of speech and volume were more critical than in a face-to-face setting.

Further, minor differences were found in the adherence to conversation rules, as there were sometimes more interruptions and fewer exchanges of words in a telesetting. In addition, a more frontal communication style was reported in the telesetting compared to the face-to-face setting. In some cases, however, the conversations were also described as more open and personal than face-to-face. Significant changes occurred in questioning behavior and were explained by the direct effects on the necessary self-management in telesetting. The interview techniques were almost identical to those used in the face-to-face setting. However, individual techniques such as action-accompanying speech, paraphrasing, active listening, and specific questioning techniques became more important.

The interactions in the telesetting were easy to implement and essentially presented as identical to face-to-face, for example, when establishing and maintaining a therapeutic relationship. Technical hurdles such as making direct eye contact more difficult had a minor impact on the interactions. Nevertheless, there were some specific changes in the area of interactions. Overall, the home environment seemed to make patients more self-confident, whereby patients were sometimes perceived as more open and talkative in their interactions. Fundamental changes were also described in the relationship between patients and relatives in the telesetting. Relatives were sometimes described as assistants and co-therapists. Increased participation of relatives was also described in telesetting compared to presence, which enabled an increased flow of information but also led to new conflicts. Relatives gained insight into what was happening and understood processes better, but could also exert more pressure on patients. Overall, telesetting was described as a relief for relatives compared to a face-to-face setting. On the other hand, the emotional transfer in interactions was described as a significant deterioration. The lack of physicality negatively impacted interactions, and professionals described a loss of control over patients’ actions at home. Mutual interactions were sometimes described as limited, and engaging in joint action and active exchange was more difficult. The telesetting was also perceived as more sterile and lonely than face-to-face.

Another question dealt with changes at a procedural and organizational level as well as environmental aspects. The procedural design of a telesetting was essentially seen as very identical to a face-to-face setting, and the course of the units was almost similar. Fundamental changes arose in the preparation and follow-up due to increased technical effort. Diagnostics and reporting were considered more difficult, although initial interviews and medical history could be conducted well in a telesetting. However, the telesetting content differed significantly from the face-to-face setting. Counseling services and everyday orientation generally increased significantly. Furthermore, the content was adapted to the home environment, although limitations in materials and methods were mentioned here.

The most significant influence on the quality experience of telesetting was functional technology and a pronounced ability for patients to self-manage. Future research and innovations are expected to lead to further quality improvements at a procedural level. In addition, changes in institutional organization compared to a face-to-face setting became clear. Critical and negative aspects such as work intensification, lack of relief time, or financial burdens due to telesetting were discussed. At the same time, organizing appointments was perceived to be easier than in a face-to-face setting. There were also further advantages in fewer absences, less travel, and interprofessional exchange. Telesetting was described as an alternative care option to face-to-face care and, in some cases, was also seen as a substitute for home visits. The advantages of low-threshold access to telesetting were formulated, particularly in crises or in the event of illness circumstances such as immobility, poor weather conditions, lack of relatives, or a very rural place of residence. In addition, higher treatment continuity was achieved than in a face-to-face setting. High acute phases of illness and chronic illnesses could be better managed in telesetting, which sometimes prevented hospital stays. Furthermore, new possibilities were described through telesetting, particularly in aftercare or prevention. The provision of telesetting also had an economic impact. For example, additional business security was formulated through an expanded clientele. However, telesetting does not yet appear to be widely established, as there was a sharp decline in telesetting units after the pandemic. In the future, a combination of tele- and face-to-face settings was described as very advantageous, and further growth in telesettings was assumed.

Environmental factors also changed compared to face-to-face settings. For example, the importance of discussing the patient's home setting well in advance increased, and the professionals recommended a professional setting in the professional premises. Technical aspects became much more critical compared to a face-to-face setting. A high usability level was crucial to creating an inclusive and participatory offer. Modern and powerful hardware was recommended for this. Expanding technical and digital skills, which could be used in many other areas of life, was seen as an advantage for everyone involved. Disadvantages included increased commitment and dependence on media. In addition, necessary changes in the framework conditions were discussed, especially concerning curricula and creating a secure legal framework. Aspects of high importance in telesetting were also of further research interest. It became clear that several general personal factors are more important for the success of telesetting, and that applies to both professionals and patients. First and foremost, digital and technical skills were mentioned here, which were seen as both a barrier and a resource. In connection with intrinsic motivation and personal attitude, this could be decisive for the acceptance and success of a telesetting and was therefore determined in advance. Such clarification is also recommended in the existing literature.^9,57,58^ In addition, specific personal factors were described for patients and professionals that were considered crucial for a successful telesetting and were different from a face-to-face setting. For patients, the individual manifestation of symptoms and resources was considered more decisive for telesetting than the diagnosis. For example, good body awareness was emphasized as particularly important, as physical reactions and sensations had to be well described to avoid risks or even damage through independent training in telesetting. Another change was seen in the self-management of patients, which was considered extremely important in telesetting and represented a significant difference from the face-to-face setting. For professionals, a change in their understanding of their professional role was emphasized, significantly influencing the perception of telesetting. Overall, the proportion of coaching and counseling in telesetting rose sharply, which had to match the individual professional self-image. There were minor changes in therapeutic behavior, with calm and structure described as more important. The professionals also stated that telesetting was perceived as more strenuous than a face-to-face setting, mainly due to more intensive observation and the need for increased concentration in the telesetting.

## Conclusions

The subjective experience of telesetting differs from a face-to-face setting in many respects. Many aspects mentioned in the interviews describe a specific process with exceptional communication and interaction in a telesetting compared to a face-to-face setting. At the same time, many factors are not yet sufficiently taught in training. Hence, acquiring specific knowledge about telesetting seems essential to developing role behavior and personal attitudes towards telesetting and increasing the acceptance and dissemination of digital therapy and care. This needs to be addressed as early as the training phase, and appropriate communicative, situational, technical, and methodological knowledge needs to be imparted. Curricular consideration has not yet been given to the extent that could correspond to technological development. Care situations often change more quickly than could be considered in curricula. It is precisely the teaching of such fundamental aspects that can promote the further spread of telecommunications. In addition, this study revealed the importance of administrative support and staff training in introducing digital care structures. Additional expertise and specialists are needed to support both sides, especially in technology and methods. Specific methods and exercises for telesetting should also be developed in the future to increase quality and quantity. It is essential to take full account of the technical possibilities and limitations and, at the same time, address the needs of patients so that the new content is as inclusive and participatory as possible. In addition to targeted funding for research projects, innovative methods are also needed here geared toward the digital audio-visual possibilities in telesetting. At the same time, a comprehensive research landscape with randomized clinical study designs is required, which, above all, makes the therapeutic outcome and the effects of tele- and face-to-face settings comparable. Knowledge of these special features and further research can help professionalize teletherapy and telecare further and support disseminating telemedical approaches.

The planned recommendations for action can benefit health professionals and patients who want to try out or further professionalize this new and promising care pathway. An initial result of this qualitative study is that it would be beneficial for health professionals and patients to have their own recommendations for action.

## Limitations

The study has several limitations that should be considered when interpreting the results. Due to the small sample, the categories and results are not representative. By including health professionals from four specialist areas and patients’ perspectives, an attempt was made to establish a cross-professional consensus.

Another important aspect is the period during which the interviews were conducted during the COVID-19 pandemic. It is conceivable, for example, that the attitude of society as a whole towards video consultations was different during the pandemic than after the pandemic, but this can be refuted by similar results from earlier studies that took place before the pandemic. At the same time, the interview data collected can be considered unique, as it was collected directly in a global risk situation and, therefore, cannot be repeated. However, the results derived from this must be interpreted about this selection and attrition bias.

There were also difficulties in recruiting health professionals and patients from nursing and physiotherapy, and the catchment area was extended to Austria and Switzerland. This may also have led to a selection bias in the choice of interviewees. In addition, the recruitment of individual interview participants after the theoretical sampling that took place at the beginning was not comprehensible for the researcher due to the subsequent snowball sampling, so a selection and attrition bias of people with an affinity for technology and attitudes towards the research topic cannot be ruled out.

In addition, national and international guidelines and recommendations on teletherapy and telecare were presented during the survey period, which, despite the iterative research, could not be fully incorporated into the interview guidelines.

## Notes

1. Number of interviewed Person, Paragraph in the interview
